# The Impact of Temporally Coherent Visual Cues on Speech Perception in Complex Auditory Environments

**DOI:** 10.3389/fnins.2021.678029

**Published:** 2021-06-07

**Authors:** Yi Yuan, Yasneli Lleo, Rebecca Daniel, Alexandra White, Yonghee Oh

**Affiliations:** Department of Speech, Language, and Hearing Sciences, University of Florida, Gainesville, FL, United States

**Keywords:** audiovisual speech recognition, multisensory gain, temporal coherence, amplitude envelope, SNR

## Abstract

Speech perception often takes place in noisy environments, where multiple auditory signals compete with one another. The addition of visual cues such as talkers’ faces or lip movements to an auditory signal can help improve the intelligibility of speech in those suboptimal listening environments. This is referred to as audiovisual benefits. The current study aimed to delineate the signal-to-noise ratio (SNR) conditions under which visual presentations of the acoustic amplitude envelopes have their most significant impact on speech perception. Seventeen adults with normal hearing were recruited. Participants were presented with spoken sentences in babble noise either in auditory-only or auditory-visual conditions with various SNRs at −7, −5, −3, −1, and 1 dB. The visual stimulus applied in this study was a sphere that varied in size syncing with the amplitude envelope of the target speech signals. Participants were asked to transcribe the sentences they heard. Results showed that a significant improvement in accuracy in the auditory-visual condition versus the audio-only condition was obtained at the SNRs of −3 and −1 dB, but no improvement was observed in other SNRs. These results showed that dynamic temporal visual information can benefit speech perception in noise, and the optimal facilitative effects of visual amplitude envelope can be observed under an intermediate SNR range.

## Introduction

Speech perception is crucial to daily communication (information exchange) for normal-hearing and hearing-impaired people of all ages. The quality of speech perception can vary based on different perceptual phenomena. For instance, speech intelligibility will decrease in demanding listening situations. In their seminal work, Sumby and Pollack (1954) examined the contribution of visual factors (such as a talker’s face) to speech intelligibility in a varied speech-to-noise ratio (which is often referred to as signal-to-noise ratio, SNR, nowadays). They concluded that the visual contribution becomes more critical as the SNR is decreased. Therefore, it is essential to study human communication in the context of audiovisual speech perception (Grant and Bernstein, 2019, for a review). More importantly, as in actual daily life, studying the audiovisual benefits to speech perception in various noise environments is critical to fully understand the contribution of visual inputs to speech perception.

A wealth of behavioral studies has examined the audiovisual benefits to speech perception utilizing different levels of noises. One line of research has focused on determining the optimal speech perception threshold conferred by visual cues. MacLeod and Summerfield (1990) measured speech perception threshold for sentences in white noise low pass filtered at 10 kHz using an adaptive tracking procedure. They reasoned that noise plays a role in masking the higher-frequency components of the acoustic signal to which the visual cues of lip shape and tongue position are complementary. The noise level was fixed at 60 dBA. The starting SNR for the list was −28 dB in the audiovisual (AV) condition and −20 dB in the auditory-only (AO) condition. The average SRT benefits were 6.4 dB, which was measured based on the 50% correct of trials. Grant and Seitz (2000) also used a three-up, one-down adaptive tracking procedure targeting the 79% point on the psychometric function (Levitt, 1971) for the noise’s intensity and fixed the target at 50-dB sound pressure level (SPL). The visual benefit calculated based on their method was averaged at 1.6 dB.

Another line of research explored audiovisual benefits in terms of setting a range of SNRs or selected SNRs, to determine the level of the most pronounced audiovisual gains. O’Neill (1954) applied four different SNRs (−20, −10, 0, and 10 dB) and found that visual recognition (AV condition) was greater than nonvisual recognition (AO condition) under all four SNRs. Sumby and Pollack (1954) applied a range of SNRs from −30 to 0 dB and proposed that the visual contribution becomes more critical as the SNR decreased. Similar to O’Neill (1954), they believed that because the intelligibility is much lower in AO condition at low SNRs (for instance, −20 dB), the visual cues’ contributions are reflected in the percentage of correct responses. However, as pointed out in their own description, the stimuli used in this study were a close set of isolated spondee words, which refer to the words with two equally stressed syllables such as “Sunset” and “Toothache.” The spondees were presented to the subjects prior to the testing and as a checklist during the testing. In this case, relatively low SNR would have a higher impact with this procedure as the participants could guess the words from a small and consistent candidate pool. To eliminate the drawbacks of the closed-set testing stimuli, Ross et al. (2007a, b) applied a much larger stimulus set to find the highest gain at the intermediate SNRs. They measured the speech perception of normal subjects and schizophrenia patients at SNRs 24, 20, 16, 12, 8, 4, and 0 dB, respectively. They found that the maximum multisensory gain difference between the AV and AO speech perception occurred at the 12-dB SNR; the audiovisual gain showed an inverted U-shaped curve as a function of the SNR. A similar SNR trend was observed in the study of Liu et al. (2013). They measured audiovisual benefits with Chinese monosyllabic words in pink noise with different SNRs at −16, −12, −8, −4, and 0 dB, in behavioral recognition and event-related potential (ERP) paradigms. This behavioral study found that the maximum difference in speech recognition accuracy between the AV and AO conditions was at the −12-dB SNR, which is aligned with the highest ERP evoked that is observed at the same SNR. Taken together, these studies show that audiovisual benefits are not more significant under low SNR conditions, but instead, there was a special zone at a more intermediate SNR (such as −12 dB) where audiovisual integration results in substantial benefits.

Regardless of the various selected SNRs applied in the studies mentioned above, they all share a common visual stimulus such as actual talkers’ faces or lip movements. Lip-reading education prevails in the hearing community (Campbell and Dodd, 1980). However, according to studies, some articulatory activities cannot be detected through lip-reading (Summerfield, 1992, for a review). Given the limited articulation information conveyed by lip movements, it is important to know which visual features (for example, lip movements) truly benefit audiovisual integration in speech perception. According to the motor theory (MT, Liberman et al., 1967; Liberman and Mattingly, 1985), the authors claim that speech perception entails recovery of the articulatory gestures or the invariant neuromotor commands. Speech in noise is perceived more accurately when the speakers are seen as special language information was encoded in the lip movements (Erber, 1975). Erber (1979) synthesized an oscilloscope pattern to resemble the actual lip configurations by tracking F1 in vowels and other signals for lip width. Grant and Seitz (2000), Grant (2001) calculated the correlation between the spatial information of mouth opening, speech amplitude peaks, and different formants. They demonstrated that observation of the lips and face movements yield more phonemic-level details and improve the speech detection threshold. In the study of Jaekl et al. (2015), the researchers explored the contribution from the dynamic configural information to speech perception by applying point-light displays to a motion-captured real talker’s face. They suggested that the global processing of the face shape changes contributed significantly to the perception of articulation gestures in speech perception.

In contrast to MT, the proponents of the general auditory learning approaches (GA, Diehl and Kluender, 1989; Diehl et al., 2004) to speech perception contend that speech perception is performed by mechanisms related to all environmental stimuli. They argue that perceptual constancy is the result of the system’s general ability to combine multiple imperfect acoustic cues without recovery of the articulatory gestures. To be more specific, from our study’s perspective, the essence of lip-reading information is delivering the temporal cues of the acoustic signals, which is a shared characteristic across visual and auditory modalities (Atilgan et al., 2018). Whereas some visual stimulus may impact individual phoneme recognition (e.g., the McGurk effect; McGurk and MacDonald, 1976), when it comes to running speech, the dynamic movements of the mouth provide an analog of the acoustic amplitude envelope, which conveys the temporal information as audiovisual binding foundation cross modalities (Maddox et al., 2015; Bizley et al., 2016).

In our previous study (Yuan et al., 2020), we used an abstract visual presentation (a sphere) of the amplitude envelope cues from target sentences to assist speech perception in a fixed −3-dB SNR background noise. Significant speech performance improvements were observed with the visual analog of the amplitude envelope, without any actual face or lip movements. Our research’s central hypothesis is that dynamic temporal visual information provides benefits for speech perception, independent of particular articulation movements. Bernstein et al. (2004) used several abstract visual representations of speech amplitude envelopes, such as a Lissajous curve and a rectangle in their research. Even though their results showed a decrease in speech detection thresholds under AV conditions, they did not find greater audiovisual benefits when comparing abstract visual cues with the actual talker’s face. These results partly reflect the limitations of the test materials, which included isolated phonemic combinations. We hypothesize that it is the tracking of temporal cues of visual signals synced with auditory signals that plays a key role in the perception of continuous speech and speech intelligibility enhancement. Continuous speech tracking relies more on audio-to-visual correlation from the temporal domain, different from the single phoneme recognition tasks (Zion Golumbic et al., 2013). Therefore, our studies used speech sentences instead of phonemic combinations. In our previous study (Yuan et al., 2020), eight separately recruited subjects were tested on 30 sentences at SNRs of −1, −3, and −5 dB from both male and female speakers presented in the AO condition. Those behavioral piloting data were used to select appropriate SNRs for testing. The results indicated that an SNR of −3 dB for both female (mean = 77.03%, SD = 20.64%) and male (mean = 62.11%, SD = 28.64%) speakers yielded an appropriate level of performance to avoid ceiling and floor effects. However, in real-life settings, listeners are facing a fast-changing listening environment with various levels of noise. These findings lead us to the question of whether our previous results would hold with other SNR conditions. In the current study, we hypothesized that similar to previous research testing with word stimuli (Ross et al., 2007a; Liu et al., 2013), intermediate SNRs for optimal gain by audiovisual enhancement would also be observed on sentence-level speech perception in noise.

## Materials and Methods

### Subjects

Seventeen adult subjects, 14 female and three male adult subjects (average age 21 ± 1.6 years old), participated in the experiment. All subjects had normal audiometric hearing thresholds (air conduction thresholds ≤ 25 dB hearing level) and were screened for normal cognitive function using the Montreal Cognitive Assessment (MoCA, Nasreddine et al., 2005) with a minimum score of 29 out of 30 required to qualify (mean MoCA 28.76). All subjects were native, monolingual English speakers with normal vision. All experiments were conducted in accordance with the guidelines for the protection of human subjects as established by the Institutional Review Board (IRB) of the University of Florida, and the methods employed were approved by that IRB.

### Stimuli and Procedures

**FIGURE 1:**
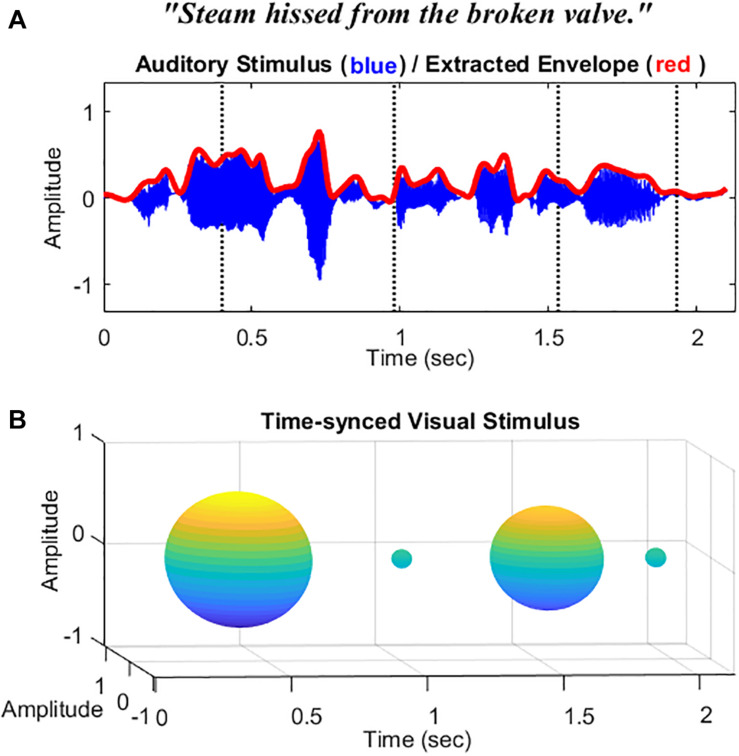
Schematic representation of the stimuli used in this study. **(A)** The waveform and the extracted temporal envelope of a sample speech sentence “Steam hissed from the broke valve.” **(B)** Sphere-shaped visual symbol synchronized with the acoustic speech amplitude envelope at time intervals 400, 950, 1,550, and 1,950 ms indicated by the dotted vertical lines.

Auditory stimuli consisted of five lists of speech sentences (each list consists of 10 sentences) from the Harvard sentences (Institute of Electrical and Electronic Engineers, 1969). Each list was recorded by male and female native English speakers (5 sentences for each speaker, 25 sentences in total for the male speaker, and 25 for the female speaker). Ten sentences were chosen for the practice section and 40 sentences for testing (20 sentences for AO condition and 20 for AV condition). An example speech sentence is described in Figure 1A. The target sentences were sampled at 44.1 kHz, and root-mean-square (RMS) matched through MATLAB (R2019a, MathWorks, Natick, MA, United States) at a fixed 65-dB SPL for presentation. Target sentences were embedded within a multi-talker babble noise with a 200-ms duration of the noise added before and after. Eight-talkers babble noise was prerecorded and normalized by MATLAB with various intensities at 72, 70, 68, 66, and 64 dB SPL. The obtained SNR was −7, −5, −3, 1, and 1 dB, respectively.

For visual stimuli in the AV condition, instead of videos of the actual talker’s face or lips, a visual analog of the amplitude envelope was applied as in our previous study (Yuan et al., 2020). Based on Yuan et al. (in press), amplitude envelopes were first extracted from the wideband target sentences and then passed through a low-pass filter (fourth-order Butterworth) with a fixed cutoff of 10 Hz. This cutoff frequency was found to be the optimal cutoff frequency cue for modulating the visual analog and getting better AV benefits than the others tested in the study (4 and 30 Hz). This parameter was also consistent with the findings from Drullman et al. (1994), which indicated that a 4–16-Hz modulation rate significantly benefits speech perception. An example envelope (red) extracted from the sentence (blue) is described in Figure 1A. A sphere was then generated based on the filtered amplitude envelope information with a fixed amplitude modulation depth at 75%. Changes in the volume of the sphere were synced with the changes in the acoustic amplitude envelope of the sentences (in isolation). See the schematic diagram of the visual stimuli in Figure 1B. The videos were rendered into 896 ^∗^ 896-pixel movies at 30 frames/s. The audio and video files were initially temporally aligned and combined as a video format in the AVS video editor (Online Media Technologies Ltd. software). For the AO condition, a video with a blank background was presented. A fixation was shown at the beginning of the videos for both conditions to alert participants to pay attention to the coming stimuli (see Supplementary Material for AO and AV example stimuli used in this study).

Speech perception tasks were conducted in a single-walled, sound-attenuated booth. All testing trials were presented through MATLAB. Audio stimuli were processed through an RME Babyface Pro sound card (RME Audio, Haimhausen, Germany) and presented through a speaker (Rokit 5, KRK Systems, Deerfield Beach, FL, United States) positioned at 0° (azimuth) in front of the listener’s head. The loudspeaker was calibrated with a Brüel & Kjær sound level meter (Brüel & Kjær Sound & Vibration Measurement A/S, Nærum, Denmark). Visual stimuli were shown on a 24-in. touch screen monitor (P2418HT, Dell, Austin, TX, United States).

For practice trials, subjects were presented with 10 audiovisual stimuli presented with clear speech. This was done to familiarize the subjects with both the visual stimuli and the auditory signals. They were then asked to type the sentence they heard into the input window, which appears once the stimulus presentation is finished. Correct answers and feedback were provided to the subject after each trial. For the testing session, 40 sentences were presented to the participant: 20 sentences in AO condition and 20 sentences in AV condition with multi-talker babble noise. At each of the SNR levels (SNR −7, −5, −3, −1, and 1 dB), four sentences were presented in the AO condition and four in the AV condition, resulting in five SNR blocks and a total of 40 target sentences. The presentation order of different SNR blocks was randomized for each participant. Target sentences with different conditions were also randomly presented within each block. Participants were asked to pay attention to the monitor until the stimulus was fully presented and then type down what they heard. Each target sentence was only presented once. We emphasized that punctuation and capitalization were not required; however, spelling was a priority. Data scoring was calculated based on complete sentences. The percentage of words accurately identified in each sentence was calculated and cross-checked by two trained research assistants in the lab. All of the scoring processes were based on our project-scoring guide. For instance, if a word is missing a phoneme or has a typo but is still clearly the same word (like photograph versus photography), it was scored as correct.

## Results

**FIGURE 2:**
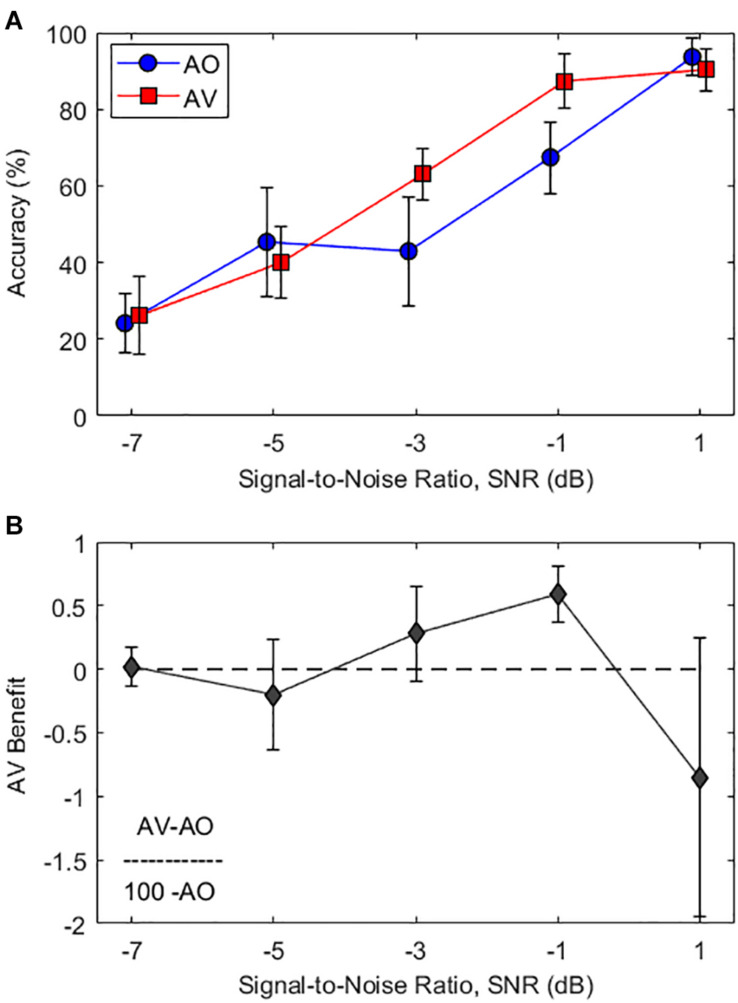
**(A)** Average word recognition accuracy results as a function of various SNR levels. The blue circle and red square symbols represent accuracy results at the audio-only (AO) and audiovisual (AV) conditions, respectively. **(B)** Average AV benefit scores as a function of various SNR levels calculated by Eq. (1) (i.e., $\frac{A\,V-A\,O}{100-A\,O}$). Error bars represent standard deviations of the mean.

Figure 2A shows the averaged word recognition accuracy as a function of various SNR levels in AO condition and AV condition. As seen in Figure 2A, the average subject responses in both the AV (red curve) and AO (blue curve) conditions demonstrate an S-shaped perceptual curve (i.e., psychometric curve). In the intermediate SNR levels between −3 and −1 dB, approximately 20% improvement of the mean word recognition accuracy was observed when synchronized visual cues were provided (AO to AV: 43 to 63% at the −3-dB SNR condition and 67–87% at the −1-dB SNR condition). No audiovisual benefit was observed in other SNRs.

A two-way repeated-measures analysis of variance (RM-ANOVA) with factors of condition (AO and AV) and SNR (−7, −5, −3, −1, and 1 dB) were employed to analyze the data, in order to see the impacts on sentence recognition accuracy (dependent variable). The results showed significant main effects of the condition (*F*_1,16_ = 52.9, *p* < 0.001, *η*^2^ = 0.768) and SNR (*F*_4,64_ = 297.1, *p* < 0.001, *η*^2^ = 0.949) and significant interaction between the condition and SNR (*F*_4,64_ = 14.7, *p* < 0.001, *η*^2^ = 0.479). *Post-hoc* pairwise comparisons using Bonferroni correction were performed to better understand the main effect of SNR. In the intermediate SNR levels between −3 and −1 dB, there were significant accuracy performance benefits in AV conditions compared with AO conditions (*p* < 0.001 for both cases). However, no significant difference was observed in other SNR conditions.

In particular, a significant interaction between condition and SNR indicates that audiovisual benefits were optimal at certain SNR ranges. The lowest (−7 dB) and the highest (1 dB) SNRs were counted as floor and ceiling effects; the intermediate zone for optimal audiovisual benefits is from −3 to −1 dB SNR. In order to demonstrate the relative amount of gain from integrating an auditory and visual cue, we applied the formula for audiovisual benefits scores from Sumby and Pollack (1954):

(1) $$A\,u\,d\,i\,o\,v\,i\,s\,u\,a\,l\,B\,e\,n\,e\,f\,i\,t\,s=\frac{A\,V-A\,O}{100-A\,O}\,\left(1\right)$$

Figure 2B displays the audiovisual benefit curve corrected for the ceiling effect as a function of SNR levels. The results show that significant audiovisual benefits were observed only at intermediate SNRs of −3 and −1 dB [*t*(16) > 3.1, *p* < 0.008 for both cases, one-sample *t*-tests]. It should be noted that there was significant audiovisual interference observed at the highest SNR of 1 dB [*t*(16) = −2.9, *p* = 0.01, one-sample *t*-tests). This very intriguing observation will be discussed in detail later.

## Discussion

We explored the optimal gain provided by audiovisual integration on speech perception in noise. An intermediate SNR zone was established at which audiovisual integration generated the greatest benefits. Our study found that, on the sentence level, the audiovisual integration benefits from temporal coherence across two modalities can be achieved optimally at the −3- and −1-dB SNR. Meanwhile, no audiovisual benefits were observed at −7 dB SNR and a significant interference between auditory and visual signals occurred at 1 dB SNR. In very early studies, it was suggested that visual contributions play more important roles than the SNR (Sumby and Pollack, 1954). Similar findings were observed in Grant and Braida’s (1991) study. When evaluating the articulation index for AV input, they tested VO, AO, and AV conditions using IEEE sentences with various SNRs (approximately from −11 to +2 dB). They found a consistent result as Sumby and Pollack (1954) that the absolute contribution of lip-reading is greatest when the auditory signal is most degraded. However, in more recent works on this topic, researchers have pointed out that for the maximum amount of audiovisual integration to be gained, a special zone of SNRs is needed. Ross et al. (2007a) found that the optimal window for maximal integration is from −24 to 0 dB SNR and the locus of the audiovisual benefit was at −12 dB SNR. Liu et al. (2013) found that audiovisual enhancement was achieved the most for both behavioral and EEG data at −12 dB SNR, aligned with the findings from Ross et al. (2007a). Taken together with our findings, a special range of SNRs was observed at which the audiovisual benefits can be gained at the optimal level on both word- and sentence-level speech perception.

More importantly, the current findings show that temporal envelope information delivered through the visual channel can serve as a reliable cue for audiovisual speech perception performance in various noise level conditions. It should be noted that our previous studies revealed that a visual analog that is temporally synchronized with the acoustic amplitude envelope (i.e., congruent condition) significantly improved speech intelligibility in noise; however, incongruent visual stimuli disrupted the integration foundation for auditory and visual stimuli (Yuan et al., 2020). Furthermore, the benefit from the congruent visual analog was optimized with specific temporal envelope characteristics of 10-Hz modulation rate and 75% modulation depth (Yuan et al., in press). In sum, the findings in a series of our studies support that the temporal characteristic of the visual inputs plays a fundamental role in audiovisual integration in speech perception and audiovisual benefits have a nonlinear relationship with the various noise levels.

Ross et al. (2007a) mentioned that their audiovisual speech perception behavioral results were not completely consistent with the interpretation of inverse effectiveness (Meredith and Stein, 1986); that is, the multisensory gain was not most significant when the unisensory input (auditory signal in this case) was weakest. In the literature, the underlying mechanism of multisensory integration has been vastly investigated, and the principles of multisensory integration (spatial, temporal, and inverse effectiveness) were wildly accepted (Meredith and Stein, 1986; Meredith et al., 1992; Wallace et al., 1993). According to the principle of inverse effectiveness, multisensory interaction at the cellular level can be superadditive when each unisensory input alone elicits a relatively weak neural discharge (Meredith and Stein, 1986). Studies of audiovisual interactions in early evoked brain activity followed the principle of inverse effectiveness (Senkowski et al., 2011). The presentation method in this study was to modulate the volume of sound presentation or the luminance of visual presentation with nonspeech stimuli, under unisensory and bisensory conditions. However, different from the electroencephalography (EEG) studies, it would be arbitrary to simply generalize the principles of multisensory integration from a single cellular response level to human behavioral studies (Stein and Stanford, 2008; Holmes, 2009; Stein et al., 2009), especially with speech perception tasks. In our present study, when the SNR was the lowest (−7 dB SNR), no audiovisual benefit was observed. From this, it is reasonable to suggest that there are minimal levels of auditory input required before speech perception can be most effectively enhanced by visual input (Ross et al., 2007a). It should be noted that our study tested only four sentences (20 words) per SNR condition, and thus, the small numbers of the sentences can lead to inappropriately characterizing audiovisual gain. Ross et al. (2007a) study explored this issue with various gain functions [gain in percent (AV-AO)^∗^100/AV; gain corrected for the ceiling effect (AV-AO)/(100-AO); gain in dB] and found that all three functions characterize the audiovisual gain well even with a small sample size (25 words per noise level). Future work will need to examine the effects of the numbers of stimuli and types of audiovisual gain functions in audiovisual speech perception with analog visual cues.

Speech perception is a continuous perceptual categorization that is context-sensitive (Holt and Lotto, 2010) rather than binary responses of categorical perception (Ma et al., 2009). It has been reported that nonspeech sounds similar in their spectral or temporal characteristics of speech signals can influence speech categorization (Holt, 2005). This finding demonstrates that general auditory processes are involved in relating speech signals and their contexts. Hence, Bizley et al. (2016) proposed that there is an early multisensory integration that may form a physiological substrate for the bottom-up grouping of auditory and visual stimuli into audiovisual objects. Based on our results, we found that a significant interference across auditory and visual modalities occurred at the highest SNR (+1 dB SNR), which means no audiovisual benefit was observed at this level of SNR. There are two possible explanations behind this phenomenon. First, when a noise in the environment was of sufficient magnitude to mask speech signals, visually delivered amplitude envelope information would contribute to a co-modulation masking release function (Hall and Grose, 1988, 1990; Moore et al., 1990) to enhance masked target auditory signals from the noise. The visual analog of the amplitude envelope itself was a complementary cue to the auditory cues. In other words, the auditory event was the primary perception source as proposed in the GA theory (Diehl and Kluender, 1989), and visual inputs provided extra assistance to the auditory signals. The additional visual channel transferred the same or a subset of the amplitude envelope information of the target signal. Therefore, the target signals were enhanced and released from the background noise. We defined this function as a bimodal co-modulation masking self-release (BCMSR, Yuan et al., in press). If the auditory signal was intelligible and unambiguous by itself (for instance, the target signal is 1 dB louder than the background noise), the co-modulation across visual and auditory signals would not show significant enhancement. In addition, a higher cognitive process may be essential in top-down attention-shifting in visual–tactile interaction (Kanayama et al., 2012) or audiovisual interaction (Bizley et al., 2016). Since the target auditory signal is clear enough, subjects might shift their attention from co-modulating the visual presentation of the amplitude envelope with target auditory signals (which were already very intelligible) to the background noise signals (multi-taker babble noise in the present study). This attention-shifting may cause significant interference in audiovisual benefits at higher SNRs (see Maddox et al., 2015; Bizley et al., 2016, for a review on divided attention tasks in multisensory integration).

## Conclusion

Speech perception frequently occurs in nonoptimal listening conditions and understanding speech in noisy environments is challenging for everyone. As a multisensory integration process, audiovisual integration in speech perception requires salient temporal cues to enhance both speech detection and tracking ability. Amplitude envelope, serving as a reliable temporal cue source, can be applied through different sensory modalities when the auditory ability is compromised. The integration across different sensory modalities also requires certain levels of SNRs to gain their optimal integration benefits. The SNRs could neither be too low, as a minimal level of auditory input being required before the speech perception can be most effectively enhanced by visual input, nor too high, as the essential top-down modulation from the higher cognitive process may shift attention from targets to background noise. Further research will focus on testing with more individualized SNR conditions. In conclusion, the temporal cue is a critical visual characteristic in facilitating speech perception in noise. Listeners can benefit from dynamic temporal visual information correlated with the auditory speech signal but do not contain any information about articulatory gestures in adverse hearing environments.

## Data Availability Statement

The original contributions presented in the study are included in the article/Supplementary Material, further inquiries can be directed to the corresponding author/s.

## Ethics Statement

The studies involving human participants were reviewed and approved by Institutional Review Board of University of Florida. The patients/participants provided their written informed consent to participate in this study.

## Author Contributions

YY and YO designed the experiments, analyzed the data, wrote the article, and discussed the results at all states. YY, YL, RD, and AW performed the experiments. All authors contributed to the article and approved the submitted version.

## Conflict of Interest

The authors declare that the research was conducted in the absence of any commercial or financial relationships that could be construed as a potential conflict of interest.
